# Expression of BRCA1 and ERCC1 as predictive clinical outcome after radiochemotherapy in patients with locoregionally moderate-advanced nasopharyngeal carcinoma

**DOI:** 10.18632/oncotarget.15565

**Published:** 2017-02-21

**Authors:** Shan Xu, Yanxin Yu, Jinfeng Rong, Defeng Hu, LiJun Zhang, Shaozhi Fu, Hongru Yang, Juan Fan, Linglin Yang, Jingbo Wu

**Affiliations:** ^1^ Department of Oncology, The Affiliated Hospital of Southwest Medical University, Luzhou 646000, P. R. China; ^2^ Department of Otolaryngology Head and Neck Surgery, The Affiliated Hospital of Southwest Medical University, Luzhou 646000, P. R. China

**Keywords:** nasopharyngeal carcinoma, ERCC1, BRCA1, induction chemotherapy, cisplatin

## Abstract

In this study, we examined ERCC1 and BRCA1 expression and clinical outcome of 201 phase-III-IV nasopharyngeal carcinoma patients who were treated with cisplatin-based induced chemotherapy and concurrent radiochemotherapy. The chemotherapy response rate of BRCA1^–^ and BRCA1^+^ patients was 73.6% and 55.8%, respectively. In addition, the chemotherapy response rate of ERCC1^–^ and ERCC1^+^ patients was 76.9% and 56.6%, respectively. In patients’ tissues, ERCC1 expression associated with BRCA1 expression. The chemotherapy response rate of BRCA1^–^ and ERCC1^–^ patients was (82.1%) and higher than that of other groups (range 52.4-73.1%). The radiochemotherapy response rate of BRCA1^–^ and ERCC1^–^ patients was higher than that BRCA1^+^ and ERCC1^+^ patients. BRCA1^–^ and ERCC1^–^ patients showed higher 3-year overall survival, failure-free survival, locoregional failure-free survival and distant failure-free survival compared to BRCA1^+^ or ERCC1^+^ patients. Moreover, the 3-year overall survival, failure-free survival and distant failure-free survival of the BRCA1^–^ and ERCC1^–^ group were higher than that of other groups. TNM stage, ERCC1 expression and the correlation between BRCA1 and ERCC1 expression seemed significant overall survival factors. In conclusion, in nasopharyngeal carcinoma patients, ERCC1 and BRCA1 may be a predictor of response to platinum-based chemotherapy and concurrent radiochemotherapy.

## INTRODUCTION

According to epidemiology, therapeutic method and prognosis, nasopharyngeal carcinoma (NPC) is a special kind of cancer in head and neck [1]. Even if it is pretty uncommon in most parts of the world, NPC is a common malignancy in China [2]. The standard dose of radiotherapy (RT) is 65-75 GY within 6-7 weeks in consideration of that NPC is very sensitive to radio. Numerous patients with their disease at a locally advanced stage were treated by RT alone, comes out the overall survival (OS) in 5 years range from 32% to 52% [3]. However, high rates of local recurrence or metastasis has tremendous influence in patients with locally advanced NPC particularly, due to they are the relevant factors of conventional RT [4, 5].

To improve survival, people with locally advanced NPC were suggested to accept the chemotherapy as an additional treatment, which is well established for metastasis, with a high level of objective response, enduring remission and in some cases of long survival [6]. Platinum-based chemotherapy is the recognized first-line treatment for metastatic NPC [7]. The function of platinum anticancer medicine will lead to the transformation of DNA structure and inhibit DNA to replicate and transcript in the end, which is based on the formation of DNA adducts [8]. Therefore, as a possible factor in the DNA repair process, the expression of genes has been studied in patients who accepted the chemotherapy with platinum. The consideration of adopting a better chemotherapeutic regimen will cause the maximization of curative effects and the minimization of adverse effects, if the efficient bio markers for chemotherapy resistance are established [9].

The excision repair cross-complementing 1(ERCC1) can be explained as a protein in the nucleotide excision repair (NER) complex is encoded by a gene, hence a group of proteins would have the ability to repair DNA damage which caused by substance forming adducts, e.g., platinum [8]. Breast cancer 1(BRCA1) is another oncosuppressor gene that had two different DNA repair systems so-called NER and double-stranded break repair (DSBR). Several studies demonstrated that high expression of BRCA1 shows a marker of platinum resistance in non-small-cell lung cancer(NSCLC) [10].

Although suggested by above studies that both ERCC1 and BRCA1 might act as efficient bio markers for NSCLC and colorectal cancer patients’ sensitivity in chemotherapy, knowledge on these biomarkers in NPC is still limited [11]. Moreover, it is currently not known if ERCC1 and BRCA1 are prognostic factors after concurrent radiochemotherapy treatment. Additionally, no analysis of previous research shows that these two factors have relevance with the treatment result and survival in the meantime. Therefore, we made the determination of expression of ERCC1 and BRCA1 in NPC patients, and did some research about the relevance between expression of these genes and clinical result of NPC.

## RESULTS

### Detection of ERCC1 and BRCA1 expression in NPC specimens

**Figure 1:**
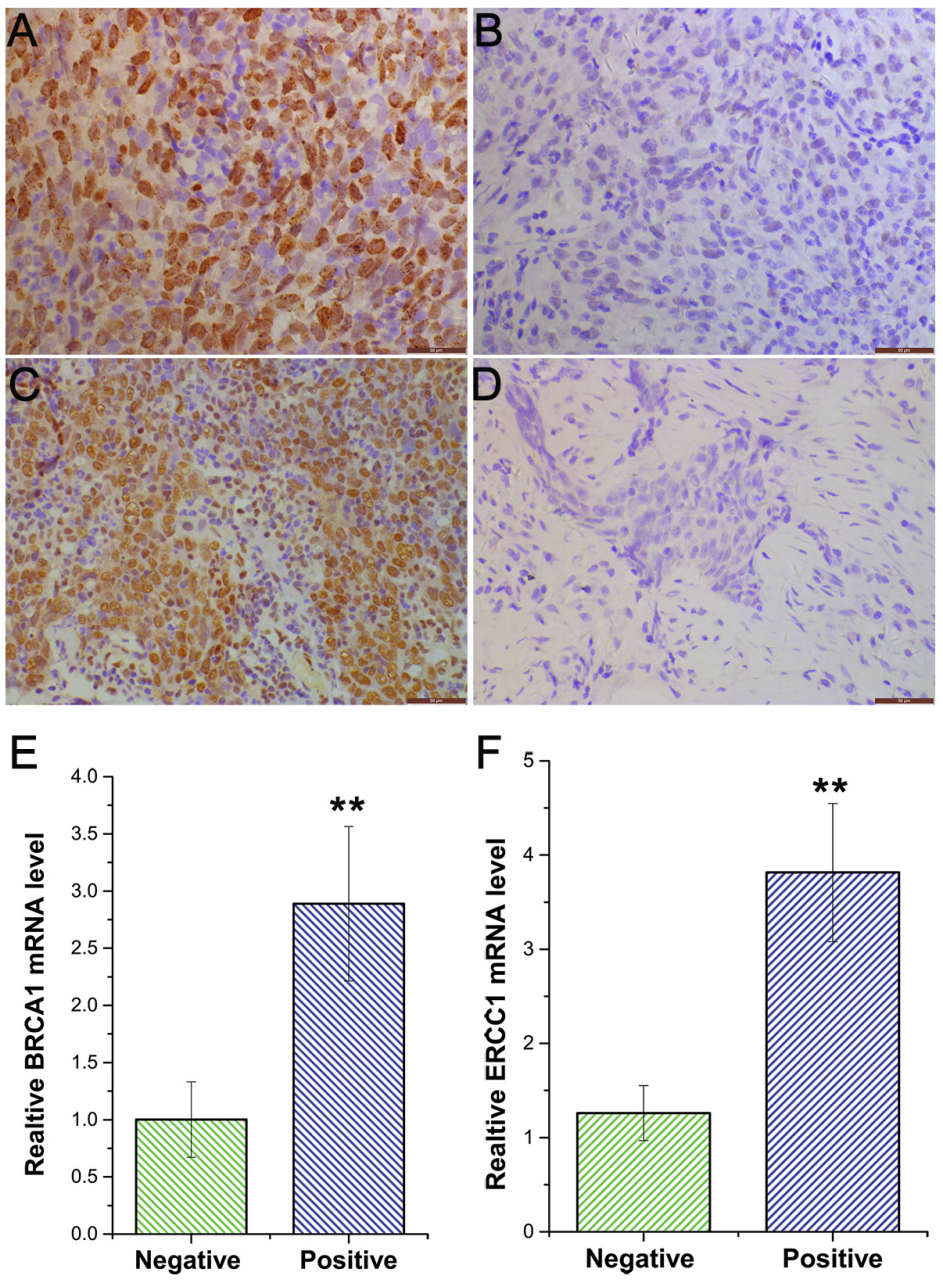
Immunostaining of BRCA1 and ERCC1 protein in nasopharyngeal carcinoma **A**. BRCA1 positive expression (X400); **B**. BRCA1 negative expression (X400); **C**. ERCC1 positive expression (X400); **D**. ERCC1 negative expression (X400). **E**. Quantification of BRCA1 expression by RT-PCR; **F**. Quantification of ERCC1 expression by RT-PCR.

Expression of BRCA1 and ERCC1 proteins was assessed by immunohistochemistry. We found that BRCA1 and ERCC1 proteins were located in the nuclei of cancer cells. BRCA1 positive expression (BRCA1^+^) was detected in tumors of 129 patients, whereas 72 patients were negative for BRCC1 negative expression (BRCA1^–^) (Figure 1). There were 136 patients with positive expression of ERCC1 (ERCC1^+^) and only 65 patients with ERCC1 negative expression (ERCC1^–^) (Figure 1). In addition, ERCC1 and BRCA1 expression in different NPC groups was analyzed by real-time quantitative (Figure 1). Quantification of relative gene expression was counted in accordance with the relative Ct method using GAPDH as a control. In the negative group, median BRCA1 mRNA expression was 1.1 (range: 0.84-1.31). In the positive group median BRCA1 mRNA expression was 2.9 (range: 2.41-4.39). Median ERCC1 mRNA expression was 1.3 in the negative group (range: 0.96-1.81), whereas in the positive group it was 3.8 (range: 2.76-4.91).

### Association of BRCA1 and ERCC1 expression with clinical pathological characteristics

Patient characteristics are summarized in Table 1. Statistical analysis showed expression of BRCA1 was associated with age (P<0.05). However, other clinical pathological parameters such as sex, pathology classification, lymph node metastasis and TNM stage had no correlations with expression of BRCA1. Similarly, there was a statistically significant difference (P<0.05) in pathology classification was found in patients with expression of ERCC1. Other clinical parameters were not statistically significant.

**Table 1:** BRCA1 expression and relationship with clinic pathological factors in nasopharyngeal carcinoma

Characteristics	BRCA1 expression				ERCC1 expression			
	N	Positive	Negative	P value^1^	N	Positive	Negative	P value^1^
SEX								
Male	132	86	46	0.757	132	94	38	0.154
Female	69	43	26		69	42	27	
Age(yr)								
≤60	178	119	59	0.037	178	118	60	0.344
>60	23	10	13		23	18	5	
WHO pathology classification								
I (keratinizing)	4	1	3	0.069	4	4	0	<0.001
II (nonkeratinizig)	159	99	60		159	96	63	
III(undifferentiated)	38	29	9		38	36	2	
T stage								
T1	10	8	2	0.275	10	5	5	0.547
T2	76	43	33		76	50	26	
T3	71	47	24		71	49	22	
T4	44	31	13		44	32	12	
Lymph node metastasis								
N0	24	15	9	0.952	24	18	6	0.190
N1	63	40	23		63	37	26	
N2	77	50	27		77	57	20	
N3	37	24	13		37	23	14	
TNM stage AJCC group (6th ed.)								
III	123	75	48	0.291	123	86	37	0.440
IV	78	54	24		78	50	28	

^1^Statistical analysis was estimated by χ2 test, and P < 0.05 was considered statistically significant; WHO, World Health Organization; AJCC, American Joint Committee on Cancer.

### Correlation between BRCA1 and ERCC1 expression in patients with nasopharyngeal carcinoma

Table 2 shows a significant correlation between BRCA1 and ERCC1 expression in patients with NPC (Spearman's test, r=0.348, P<0.05). All of the 201 patients included 103 patients were BRCA1^+^ and ERCC1^+^, 39 patients were BRCA1^–^ and ERCC1^–^, 33 patients were BRCA1^–^ and ERCC1^+^ and 26 patients were BRCA1^+^and ERCC1^–^.

**Table 2:** The correlation between BRCA1 and ERCC1 expression

	BRCA1 (+)	BRCA1(-)	R	P^1^
ERCC1 (+)	103	33	0.348	0.000
ERCC1 (-)	26	39		

^1^Statistical analysis was estimated with Spearman correlation analysis, and P < 0.05 was considered statistically significant.

### Association of ERCC1 and BRCA1 expression with prognosis of patients with NPC after chemotherapy and concurrent chemoradiotherapy

#### Short-term outcomes

Of the 201 cases included in this study, the short-term outcomes in primary NPC and cervical lymph node after chemotherapy treatment are shown in Table 3. BRCA1^–^ patients had a higher response rate compared to BRCA1^+^ patients, P=0.013. Similarly, ERCC1^–^ patients benefited more from chemotherapy than ERCC1^+^ patients. Chemotherapy in the ERCC1^–^ group had a higher response rate compared to the ERCC1^+^ group, P=0.005. In addition, in primary NPC, BRCA1^–^ and ERCC1^–^ patients had the highest response rate compared to patients that were BRCA1^+^ and ERCC1^+^, BRCA1^–^ and ERCC1^+^ and BRCA1^+^ and ERCC1^–^, P=0.005. In cervical lymph nodes, the efficacy of chemotherapy in the BRCA1^–^ group was higher than in the BRCA1^+^ group (P=0.036). Moreover, the efficacy of chemotherapy in the ERCC1^–^ group was significantly higher from that in the ERCC1^+^ group (P=0.019). In cervical lymph nodes, patients that were BRCA1^–^ and ERCC1^–^ had a higher response rate compared to patients that were BRCA1^+^ and ERCC1^+^, BRCA1^–^ and ERCC1^+^ and BRCA1^+^ and ERCC1^–^ (P=0.032).

**Table 3:** Association of BRCA1 and ERCC1 and the effect of chemotherapy in primary nasopharyngeal carcinoma and cervical lymph node

Expression		Response					
In primary		N	CR+PR	SD+PD	RR (%)	X^2^	P^1^
BRCA1							
+		129	72	57	55.8	6.224	0.013
-		72	53	19	73.6		
ERCC1							
+		136	77	59	56.6	7.795	0.005
-		65	50	15	76.9		
BRCA1	ERCC1						
+	+	103	54	49	52.4	12.840	0.005
-	-	39	32	7	82.1		
+	-	26	19	7	73.1		
-	+	33	23	10	69.6		
**In cervical lymph node**							
BRCA1							
+		129	86	43	66.6	4.387	0.036
-		72	58	14	80.5		
ERCC1							
+		136	89	47	65.4	5.496	0.019
-		65	53	12	81.5		
BRCA1	ERCC1						
+	+	103	62	41	60.1	8.831	0.032
-	-	39	33	6	84.6		
+	-	26	20	6	76.9		
-	+	33	24	9	72.7		

^1^Statistical analysis was estimated by χ2 test, and P < 0.05 was considered statistically significant; CR, complete response; PR, partial response; SD, stable disease; PD, progressive disease, RR, response rate.

Short-term outcomes of patients after radiochemotherapy treatment are shown in Table 4. In primary NPC and cervical lymph nodes, no significant differences could be detected in BRCA1 or ERCC1 expression (P>0.05). Furthermore, in primary NPC and cervical lymph nodes, no significant correlation was detected between BRCA1 and ERCC1 expression (P values were 0.218 and 0.338, respectively).

**Table 4:** Association of BRCA1 and ERCC1 and the effect of radiochemotherapy in primary nasopharyngeal carcinoma and cervical lymph node

Expression		Response					
In primary		N	CR	PR	RR(%)	X^2^	P^1^
BRCA1							
+		129	106	22	82.1	2.071	0.209
-		72	65	7	90.2		
ERCC1							
+		136	113	23	83.1	0.715	0.532
-		65	57	8	87.6		
BRCA1	ERCC1						
+	+	103	84	19	81.5	4.243	0.218
-	-	39	37	2	94.8		
+	-	26	23	3	88.4		
-	+	33	28	4	84.8		
**In cervical lymph node**							
BRCA1							
+		129	118	11	91.4	1.356	0.387
-		72	69	3	95		
ERCC1							
+		136	123	13	90.4	3.437	0.108
-		65	64	1	98.4		
BRCA1	ERCC1						
+	+	103	91	12	88	3.370	0.338
-	-	39	38	1	97		
+	-	26	24	2	92		
-	+	33	31	2	93		

^1^Statistical analysis was estimated by χ2 test, and P < 0.05 was considered statistically significant; CR, complete response; PR, partial response; RR, response rate.

#### Long-term outcomes

**Figure 2:**
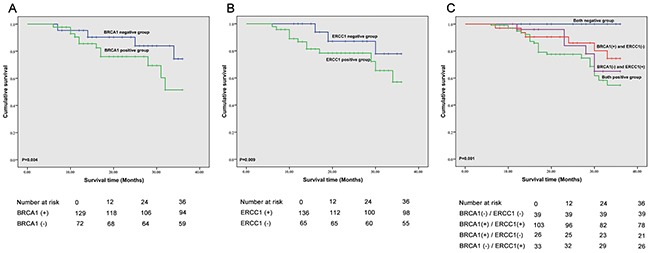
Correlation of BRCA1 and ERCC1 expression and overall survival **A**. Correlation between BRCA1 expression and overall survival (OS); **B**. Correlation between ERCC1 expression and OS; **C**. Correlation between BRCA1 and ERCC1 expression and OS.

**Figure 3:**
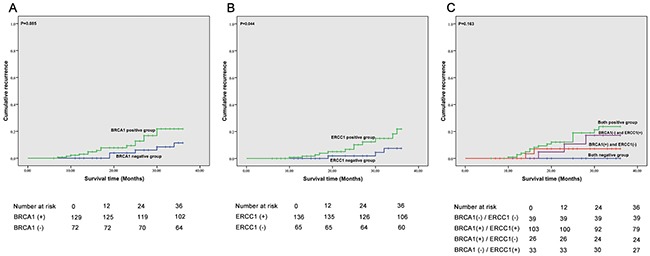
Correlation between expression of BRCA1 and ERCC1 recurrence **A**. Correlation between BRCA1 expression and recurrence; **B**. Correlation between ERCC1 expression and recurrence; **C**. Correlation between BRCA1 and ERCC1 expression and recurrence.

**Figure 4:**
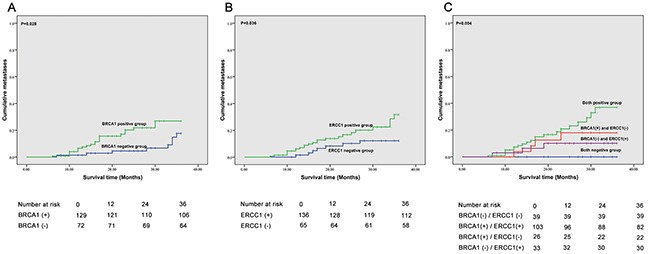
Correlation between BRCA1 and ERCC1 expression and metastases **A**. Correlation between BRCA1 expression and metastases; **B**. Correlation between ERCC1 expression and metastases; **C**. Correlation between BRCA1 and ERCC1 expression and metastases.

The 3-year overall survival (OS), failure-free survival (FFS), locoregional failure-free survival (LR-FFS), distant failure-free survival (D-FFS) and median survival time (MST) of BRCA1 and ERCC1 expression groups are shown in Table 5 and Figure 2, Figure 3 and Figure 4. We found that ERCC1^+^ or BRCA1^+^ patients had significantly poorer prognoses than patients with negative expression of ERCC1 and BRCA1. More specifically, patients that were BRCA1^–^ had a better 3-year cumulative survival than patients that were BRCA1^+^ (P=0.004). Moreover, a significant correlation was observed in the negative/positive expression of BRCA1 in 3 year D-FFS (P=0.028). Unfortunately, no significant differences were found in 3-year LR-FFS between BRCA1^–^ and BRCA1^+^ groups (P =0.085). In BRCA1^–^ patients, the MST was 34.3 months (95% CL: 33.9 - 35.3), whereas in BRCA1^+^ patients MST was 30.8 months (95% CL: 28.8 - 31.9).

**Table 5:** The correlation between BRCA1 and ERCC1 expressions in long-term outcomes

Expression		3-year OS (%)	P^1^ value	3-year FFS (%)	P^1^ value	3-year LR-FFS (%)	P^1^ value	3-year D-FFS (%)	P^1^ value	MST (Months)
BRCA1										
+		72.8(94/129)	0.004	73.6(95/129)	0.048	79.8(102/129)	0.085	82.2(106/129)	0.028	30.8(28.8-31.9)
-		81.9(59/72)		83.3(60/72)		88.9(64/72)		88.9(64/72)		34.3(33.9-35.3)
ERCC1										
+		77.8(98/136)	0.009	72.7(99/136)	0.036	77.9(106/136)	0.044	82.4(112/136)	0.036	31.6(29.8-32.6)
-		84.6(55/65)		87.6(57/65)		92.3(60/65)		89.2(58/65)		34.8(32.7-35.8)
BRCA1	ERCC1									
+	+	75.7(78/103)	0.001	62.1(64/103)	0.002	76.6(79/103)	0.163	79.6(82/103)	0.004	30.5(28.5-31.7)
-	-	100(39/39)		100(39/39)		100(39/39)		100(39/39)		36(36-36)
+	-	81.0(21/26)		84.6(22/26)		92.3(24/26)		84.6(22/26)		34.6(32.4-35.1)
-	+	78.7(26/33)		72.7(24/33)		81.8(27/33)		90.9(30/33)		32.2(29.5-34.9)

^1^: Statistical analysis was estimated with log-rank, and P < 0.05 was considered statistically significant; OS: overall survival; FFS: failure-free survival; LR-FFS: locoregional failure-free survival; D-FFS: distant failure-free survival.

Similarly, the 3-year cumulative survival in ERCC1^–^ patients was higher than in ERCC1^+^patients (P = 0.009). In addition, a statistically significant difference was observed in 3-year D-FFS between ERCC1^–^ and ERCC1 ^+^ patients (P =0.036). In addition, the 3-year LR-FFS in ERCC1^–^ patients was significantly different from that observed in ERCC1^+^ patients (P=0.044). In ERCC1^–^ patients, the MST was 34.8 months (95% CL: 32.7 - 35.8) and in ERCC1^+^ patients, MST was 31.6 months (95% CL: 29.8 - 32.6).

The correlation between BRCA1 and ERCC1 expression and 3-year OS, FFS, LR-FFS, D-FFS and MST are summarized in Table 5, Figure 2, Figure 3 and Figure 4. We found that patients with BRCA1^–^ and ERCC1^–^ expression had a significantly better prognosis than patients that showed BRCA1^+^ and ERCC1^+^ expression, BRCA1^–^ and ERCC1^+^expression, or BRCA1^+^ and ERCC1^–^ expression. The 3-year cumulative survival was significantly higher ERCC1^–^ /BRCA1^–^ patients than in BRCA1^+^ /ERCC1^+^ patients, BRCA1^+^ /ERCC1^–^ patients and BRCA1^–^ /ERCC1^+^ patients (P = 0.001). The 3-year D-FFS was also significantly different in patients with BRCA1^–^ and ERCC1^–^, BRCA1^+^ and ERCC1^+^, BRCA1^+^ and ERCC1^–^ and BRCA1^–^ and ERCC1^+^ (P = 0.004). However, no significant differences were observed between BRCA1 and ERCC1 expression in 3-year LR-FFS (P>0.05). The MST was 36 months for BRCA1^–^ and ERCC1^–^ patients (95% CL: 36.0 - 36.0), 30.5 months for BRCA1^+^ and ERCC1^+^ patients (95% CL: 28.5 - 31.7), 34.6 months for BRCA1^+^ and ERCC1^–^ patients (95% CL: 32.4 - 35.1) and 32.2 months for BRCA1^–^ and ERCC1^+^ patients (95% CL: 29.5 - 34.9).

In this study, we performed Cox regression for univariate and multivariate analyses (Table 6). Univariate analyses shown that BRCA1 expression, ERCC1 expression, and TNM stage were related to overall survival (P=0.042, 0.014 and 0.024, respectively). In addition, we found that the correlation between BRCA1 and ERCC1 expression was also related to overall survival (P<0.05 in all cases). Multivariate analyses further indicated that ERCC1 expression, TNM stage and the correlation between ERCC1 and BRCA1 expression were prognostic factors for OS (P < 0.05 in all cases).

**Table 6:** Univariate and multivariate analyses of survival (Cox regression)

Variables	Survival	
	HR (95% CI)	P value
**Univariate analysis**		
Sex	0.614(0.223-1.692)	0.346
Male/female		
Age (yr)	1.395(0.408-4.769)	0.595
≤60/> 60		
WHO pathology classification	0.382(0.110-1.330)	0.131
Keratinizing		
Nonkeratinizig		
undifferentiated		
T stage	2.823(0.943-8.447)	0.064
T1-T2/T3-T4		
Lymph node metastasis	0.502(0.168-1.505)	0.219
N0/N1-3		
TNM stage	2.877(1.147-7.213)	0.024
III/IV		
BRCA1 expression	3.141(1.041-9.472)	0.042
Negative/positive		
ERCC1 expression	6.327(1.457-27.483)	0.014
Negative/positive		
Correlation between BRCA1 and ERCC1		
BRCA1 (−) and ERCC1 (−)	Reference group	
BRCA1 (+) and ERCC1 (−)	4.126 (1.268-13.428)	0.019
BRCA1 (−) and ERCC1 (+)	4.297 (1.363-13.545)	0.013
BRCA1 (+) and ERCC1(−)	4.410 (1.543-12.604)	0.006
**Multivariate analysis**		
TNM stage	2.923(1.155-7.393)	0.024
III/IV		
BRCA1 expression	1.743(0.557-5.456)	0.340
Negative/positive		
ERCC1 expression	5.582(1.233-25.274)	0.026
Negative/positive		
Correlation between BRCA1 and ERCC1		
BRCA1 (−) and ERCC1 (−)	Reference group	
BRCA1 (+) and ERCC1 (−)	3.582 (1.098-11.6.85)	0.034
BRCA1 (−) and ERCC1 (+)	4.786 (1.516-15.107)	0.008
BRCA1 (+) and ERCC1(+)	5.157 (1.798-14.792)	0.002

HR: Hazard ratio; CI: Confidence interval.

## DISCUSSION

NPC is a malignant tumor, with a approximate incidence of 20/100,000 in China [12]. Because over half of the NPC patients fall in the International Union Against Cancer Stage III or IV [13], defining the optimal treatment for patients with NPC is of utmost importance. Induction chemotherapy combined with radiotherapy is one of the most significant strategies for patients with advanced NPC [13–17]. Platinum-based induction chemotherapy response rate and OS differ between NPC patients, which may be due to the fact that patients may have developed drug resistance and toxicity. Therefore, it is necessary to decide that the induction chemotherapy which based on platinum could be good for what kind of patients. Biomarkers that could help diagnose NPCs may assist in identifying the most appropriate forms of chemotherapy and radiochemotherapy for NPCs.

In our work, we investigated whether BRCA1 and ERCC1 could act as biomarkers. In addition, we presented separate analyses for ORR of primary tumor and neck nodes based on BRCA1 and ERCC1 expression in primary NPC in order to assess whether or not the different treatments affect the primary tumor and the metastatic sites. Currently, treatment decisions are usually based on immunohistochemistry date of the primary tumor. Some clinical researches provided the suggestions that the status of some bio markers may alter between the primary tumor and the corresponding distant metastatic sites, including epidermal growth factor receptor (EGFR), multidrug resistance (MDR), and HER-2 [18–20]. This might be the reason that the genes in the tumor are deformed, for instance, the deficiency of chromosomal or gene silencing, and in some cases, the loss of expression in metastatic cells [21]. This biological phenomenon would explain the resistance of tumor to specific bio markers in a way, so called targeted antibody therapy if it is confirmed, while most have been still investigated somehow.

We found that BRCA1 and ERCC1 positive-expression are associated with poor prognosis in NPCs. We found that ERCC1 expression correlates with pathology classification, and that ERCC1^–^ patients benefit more from platinum-based chemotherapy than ERCC1^+^. This suggests that there is a inversely relation between ERCC1 expression and the objective response to platinum-based induction chemotherapy. This may be due to the fact that platinum-based chemotherapy enters the cells and binds to DNA, which then forms a platinum–DNA adduct, and holds back cellular proliferation and transcription through nucleotide excision repair (NER) pathway, and may lead to tumor cell death. ERCC1 accounts for most platinum–DNA adduct repairs. Furthermore, patients who received radiochemotherapy had excellent efficacy (CR were about 90% in all cases) in primary NPC and cervical lymph nodes, which makes it challenging to predict the therapeutic effect between ERCC1^–^and ERCC1^+^ patients.

The single factor analysis (Kaplan–Meier method) showed ERCC1 expression was associated with OS, FFS, LR-FFS, and D-FFS. The Cox regression analysis demonstrated that ERCC1 expression was a significant factor for OS. These findings are consistent with previous reports [22–26], and confirm that ERCC1 may be a predictor for the prognosis of NPC. Hui and Koh [27, 28] did not show clinical impact of ERCC1 in NPC patients treated with platinum-based induction. The abnormal situation was caused possibly because of the two different treatment methods that were used in the two researches. In Hui and Koh's study, NPCs undergo concurrent-adjuvant chemoradiotherapy or radiotherapy alone. However, the NPCs in our work received induction chemotherapy + concurrent-adjuvant chemoradiotherapy.

We found out that BRCA1 expression correlates with age and that expression is negative correlated to the objective response of platinum-based induction chemotherapy similar to that of ERCC1. This could be due to the fact that BRCA1 is also a key role in the NER pathway. The result found in NPC corresponded to gastric cancer and bladder cancer [29, 30]. After concurrent chemoradiotherapy, no significant differences were found in primary NPC and cervical lymph node between BRCA1^–^ and BRCA1^+^ patients (CR about 90% in all cases). In a subsequent study, the Kaplan–Meier method showed that BRCA1 expression was associated with OS, FFS and D-FFS, but not LR-FFS. We inferred that BRCA1 expression has an impact on the chemotherapy response on micrometastatic spread when the NPC was diagnosed at the very beginning [23], therefore NPC patients had different OS and D-FFS without different LR-FFS. The univariate analysis confirmed the association between BRCA1 expression and OS. Multivariate analysis, however, did not support this association. This may be due to the fact that the number of patients in this work was relatively small. If the number of patients were increased, the association between BRCA1 expression and OS would possibly reach significance.

In this study, we first describe the negative correlation between BRCA1 and ERCC1 expression (R= 0.348, P <0.01). We found that patients with negative expression of BRCA1 and ERCC1 have a better therapeutic outcome after chemotherapy and concurrent radiochemotherapy than patients with both positive expression of BRCA1 and ERCC1, BRCA1^+^ and ERCC1^–^, and BRCA1^–^ and ERCC1^+^. This may be due to the synergistic effect between of BRCA1 and ERCC1. Kaplan–Meier survival analysis demonstrated that patients with negative expression of both BRCA1 and ERCC1 had a better 3-year OS, FFS and D-FFS (P < 0.05 in all cases). Univariate and multivariate analysis also demonstrated a statistically significant difference between BRCA1 and ERCC1 expression and OS, indicating a close correlation between BRCA1^–^ and ERCC1^–^ and OS. These factors support our hypothesis that BRCA1^–^ or ERCC1^–^ patients benefitted more from chemotherapy and had a better prognosis.

Although the results are promising, there are few limitations to our study. First, this work lacks a validation cohort, hence future studies need to confirm our results. Second, the study groups used were relatively small, with a limited number of patients, and rather short-term following up. Therefore, large sample multicenter studies will be required to validate our data set.

## MATERIALS AND METHODS

### Subjects

A total of 201 patients were enrolled at the Affiliated Hospital of SOUTHWEST Medical University between February 2010 and February 2012. Follow up was until until February 2015. Inclusion criteria included the following: biopsy proven stage III to IV NPC according to the American Joint Committee on Cancer (AJCC) Cancer Staging Manual (sixth edition) [31]; no radiotherapy or chemotherapy before biopsy; no major organ dysfunction; no history of other malignancies; age >18 years. Patients with a prior history of malignancy, pregnancy, contraindications for chemotherapy, or a history of chemotherapy, radiotherapy, or surgery were excluded from the study. All samples were collected by biopsy via nasopharyngoscopy. Informed consent was obtained from all patients before conducting the study.

### Study design

All patients were analyzed using a spiral chest CT scan, chest X ray, bone scan and abdominal ultrasonogram. In addition, patients received enhanced MRI scanning in the nasopharynx and neck area in order to delineate their primary NPC and neck lymph node metastases for appraisal before treatment. Blood routine, electrocardiogram, biochemical, and plasma electrolytes were determined to eliminate chemotherapeutic contraindication. Eligible patients first received intravenous platinum-based chemotherapy, when finishing their chemotherapy, patients received enhanced MRI scanning in the nasopharynx and neck area to estimate the curative effect. Then they treated with concurrent chemoradiotherapy, when finishing their chemoradiotherapy, patients once again to received enhanced MRI scanning in the nasopharynx and neck area to estimate the curative effect (Figure 1). The treatment regimens included 80mg/m^2^ cisplatin on day 1 and 5-FU 1000 mg/m^2^ on day 2, 3 and 4. Chemotherapy cycles were administrated every 4 weeks for a maximum of 2 cycles. After finishing their chemotherapy, patients received concurrent chemoradiotherapy. This treatment regimen included radiation dose with 66-70Gy (2.2Gy per treatment) for primary NPC and nodal metastasis, radiation dose with 44-64Gy (1.6-2.0Gy per treatment) for bilateral neck, and 40mg/m^2^ cisplatin per week.

### Gene expression analysis by real-time quantitative PCR

Specimens were pulverized by pulp refiner under Trizol reagent (Invitrogen). RNA was extracted with Trizol reagent and dissolved in DEPC water. Total RNA were reverse transcribed with RevertAid™ First Strand cDNA Synthesis Kit (Fermentas) for generation of cDNA. Gene expression for ERCC1, BRCA1, and GAPDH (internal reference gene) were performed using RT-PCR. The sequences of the primers used were as follows: BRCA1 Forward 5′-GTCCAAAGCGAGCAAGAG-3′, Reverse 5′-CTGTGCCAAGGGTGAATG-3; ERCC1 Forward 5′-GATGAGGTCCCTCCTGGAGTGG-3′, Reverse 5′-AGATGGCATATTCGGCGTAGGTC-3; GAPHD (internal reference gene) Forward 5′-CATGAGAAG TATGACAACAGCCT-3′, Reverse 5′-AGTCCTTCC ACGATACCAAAGT-3; GAPHD was used as an endo-genous control and data obtained were represented as 2-DDCT [32]. The amplification parameters consisted of 40 cycles at 94 °C for 40 s, annealing at different temperature for different gene for 40 s, 72 °C for 50 s. The threshold cycle (CT) data was determinate using default threshold settings. The CT is defined as the fractional cycle number at which the fluorescence passes the fixed threshold.

### Immunohistochemistry

ERCC1 and BRCA1 expression were examined immunohistochemically using paraffin-embedded tissues. In brief, 3-μm-thick tissue sections were heated in 6.5 mmol/L citrate buffer (pH 6.0) at 100°C for 28 min, and incubated with antibodies directed against ERCC1 or BRCA1 (1:200 dilution). Immunostaining was performed using the DAKO En-Vision System (Dako Diagnostics, Zug, Switzerland). In the negative control group, the primary antibody was replaced by PBS. Expression was scored by two independent experienced pathologists. Each sample was graded according to intensity and extent of staining. The intensity of staining was scored as 0 (no staining), 1 (weak staining), and 2 (strong staining). The extent of staining was based on the percentage of positive tumor cells: 0 (no staining), 1 (1–25%), 2 (26–50%), 3 (51–75%), and 4 (76–100%). These two scores were added together for a final score. A case was considered negative if the final score was 0 or 1 (−) or 2 or 3 (±), and positive if the score was 4 or 5 (+) or 6 or 7 (++). In the majority of cases, the two examiners provided consistent results. Any inconsistencies were resolved by discussion to achieve a consensus score.

### Clinical endpoints and statistical analysis

We followed reporting recommendations for tumor marker prognostic studies (REMARK) guidelines in our study. Complete remission and partial remission were defined as responsive, stable disease and progressive disease was defined as non-responsive. FFS was defined as the time from the start of chemotherapy or concurrent radiochemotherapy to tumor recurrence, metastasis or dying of the patient, whereas OS was defined as the time from the start of chemotherapy or concurrent radiochemotherapy to dying of the patient or last follow-up.

ERCC1 and BRCA1 expression as well as clinical variables of chemotherapy were evaluated using the X^2−^test. The correlation between BRCA1 and ERCC1 expression was evaluated using Spearman correlation analysis. The cumulative recurrence, cumulative metastasis and survival probability were estimated using Kaplan–Meier analysis and differences were calculated by log-rank test. Prognostic factors for survival were determined using Cox regression analysis. All P values were two-sided; P < 0.05 was considered significant. Statistical analyses were performed using SPSS 17.0 software.
